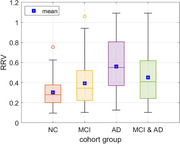# A simple measure of voluntary control of breathing is an effective physio‐marker for differentiating MCI from mild AD patients and MCI/AD patients from cognitively normal controls

**DOI:** 10.1002/alz.092034

**Published:** 2025-01-09

**Authors:** Vasilis Marmarelis, Helena C Chui, Sandra A Billinger, Elizabeth B Joe, Dae Shin, Suhaib Hashem, Jasmin Rizko, Emily Hazen, Danilo Cardim, Rong Zhang

**Affiliations:** ^1^ Department of Biomedical Engineering, University of Southern California, Los Angeles, CA USA; ^2^ Department of Neurology, Keck School of Medicine, University of Southern California, Los Angeles, CA USA; ^3^ University of Kansas Alzheimer's Disease Research Center, Fairway, KS USA; ^4^ University of Southern California, Los Angeles, CA USA; ^5^ Kansas University Medical Center, Kansas City, KS USA; ^6^ UT Southwestern Medical Center, Dallas, TX USA; ^7^ UTSW Medical Center/IEEM, Dallas, TX USA

## Abstract

**Background:**

Control of breathing is known to be adversely affected by cognitive impairment, often associated with sleep apnea or disordered breathing during sleep in MCI/AD. The origin of this disorder is thought to be in the dysfunction of the respiratory control centers of the brainstem or in the impaired afferent signaling from cortical regions. Continuous breathing data were collected in a multi‐center study in Los Angeles (USC), Kansas City (KUMC) and Dallas (UT‐SWMC), and used to compute respiratory rate variability (RRV) to test the hypothesis that voluntary control of breathing is impaired in MCI and mild AD patients relative to cognitively normal controls, and whether this impairment is more severe in mild AD than MCI patients.

**Method:**

The collected continuous breathing data were used to compute the RRV as the standard deviation of breath‐to‐breath respiratory rate variations during metronome‐guided slow‐paced breathing (6 breaths per minute) over 5 minutes under supine resting conditions for each of 61 MCI, 32 mild AD patients and 78 age/sex‐matched cognitively normal controls. The computed RRV was used to quantify and compare the voluntary control of breathing for all participants.

**Result:**

The resulting p‐values of mean‐difference t‐tests were:

• Controls vs. MCI patients: 0.304 (0.145) vs. 0.393 (0.222), *p = 0.0039*

• Controls vs. AD patients: 0.304 (0.145) vs. 0.561 (0.258), *p = 2.4E‐06*

• Controls vs. MCI & AD patients: 0.304 (0.145) vs. 0.451 (0.247), *p = 1.7E‐06*

• MCI vs. AD patients: 0.393 (0.222) vs. 0.561 (0.258), *p = 0.0014*

The results are shown in Figure 1 as box‐whisker plots. The p‐values showed significant mean‐differences in all four cases, with comparisons against mild AD patients showing greater differentiation. The observed mean RRV values showed stratification of impairment: 0.393 for MCI vs 0.561 for mild AD, relative to 0.304 for controls.

**Conclusion:**

Quantitative analysis of a simple measure of voluntary control of breathing during metronome‐guided slow‐paced breathing under supine resting conditions showed significant mean‐differences between 61 MCI patients, 32 mild AD patients and 78 age/sex‐matched cognitively normal controls (see Figure 1), suggesting that the RRV physio‐marker has the potential to aid in the diagnosis of MCI and mild AD.